# Closed-loop recycling of lithium iron phosphate cathodic powders via citric acid leaching

**DOI:** 10.1007/s11356-024-32837-6

**Published:** 2024-03-11

**Authors:** Martina Bruno, Carlotta Francia, Silvia Fiore

**Affiliations:** 1 Department of Environment, Land and Infrastructure Engineering, DIATI, Politecnico Di Torino, Corso Duca Degli Abruzzi 24, 10129 Turin, Italy; 2 DISAT, Department of Applied Sciences and Technology, Politecnico Di Torino, 10129 Turin, Italy

**Keywords:** Citric acid, Leaching, Lithium iron phosphate, Lithium recovery, Recycling

## Abstract

Lithium recovery from Lithium-ion batteries requires hydrometallurgy but up-to-date technologies aren’t economically viable for Lithium-Iron-Phosphate (LFP) batteries. Selective leaching (specifically targeting Lithium and based on mild organic acids and low temperatures) is attracting attention because of decreased environmental impacts compared to conventional hydrometallurgy. This study analysed the technical and economic performances of selective leaching with 6%vv. H_2_O_2_ and citric acid (0.25-1 M, 25 °C, 1 h, 70 g/l) compared with conventional leaching with an inorganic acid (H_2_SO_4_ 1 M, 40 °C, 2 h, 50 g/l) and an organic acid (citric acid 1 M, 25 °C, 1 h, 70 g/l) to recycle end of life LFP cathodes. After conventional leaching, chemical precipitation allowed to recover in multiple steps Li, Fe and P salts, while selective leaching allowed to recover Fe and P, in the leaching residues and required chemical precipitation only for lithium recovery. Conventional leaching with 1 M acids achieved leaching efficiencies equal to 95 ± 2% for Li, 98 ± 8% for Fe, 96 ± 3% for P with sulfuric acid and 83 ± 0.8% for Li, 8 ± 1% for Fe, 12 ± 5% for P with citric acid. Decreasing citric acid’s concentration from 1 to 0.25 M didn’t substantially change leaching efficiency. Selective leaching with citric acid has higher recovery efficiency (82 ± 6% for Fe, 74 ± 8% for P, 29 ± 5% for Li) than conventional leaching with sulfuric acid (69 ± 15% for Fe, 70 ± 18% for P, and 21 ± 2% for Li). Also, impurities’ amounts were lower with citric acid (335 ± 19 335 ± 19 of S mg/kg of S) than with sulfuric acid (8104 ± 2403 mg/kg of S). In overall, the operative costs associated to 0.25 M citric acid route (3.17€/kg) were lower compared to 1 M sulfuric acid (3.52€/kg). In conclusion, citric acid could be a viable option to lower LFP batteries’ recycling costs, and it should be further explored prioritizing Lithium recovery and purity of recovered materials.

## Introduction

Lithium ion batteries (LIBs) represent a fundamental technology to achieve European zero emissions’ target by 2050 (European Commission 2020). The forecasted increase in the sales of passengers electric vehicles will lead to 65% rise in LIBs demand, from 330 GWh in 2021 to 550 GWh in 2022 (IEA 2023). LIBs encompass economically valuable elements and critical raw materials, with significant environmental impacts and costs associated to their mining and concern about the security of the supply chain (Farjana et al. 2019; Fu et al. 2020; Sun et al. 2019). Hence, recycling End of Life (EoL) LIBs is crucial for supplying secondary materials related to the expected increase of production demand (Zhao et al. 2022).

Among LIBs, Lithium Iron Phosphate (LFP) batteries are becoming increasingly popular in the electric transport sector, since they high stability, increased safety and lower reliance on critical raw materials (Saju et al. 2023), indeed they will exceed 30% of market share by 2030 (Wood Mackenzie 2020). However, the main bottleneck related to LFP batteries recycling is that the economic trade-off between potential revenues and recycling costs is unfavorable at full-scale (Mahandra and Ghahreman 2021). Up-to-date recycling technologies prioritize recovery of the most economically valuable elements, as Cobalt, Manganese and Nickel (Chan et al. 2021; Jantunen et al. 2022; Schiavi et al. 2021), while Lithium and Phosphorous have lower market value.

Recycling EoL LFP cathodes involves hydrometallurgy based on inorganic acids (Gerold et al. 2023; Li et al. 2022; Wang et al. 2023;), achieving leaching efficiencies between 97 and 99.9% for Lithium and 98–99% for Iron with sulfuric acid (Vieceli et al. 2021; Yang et al. 2023; Song et al. 2021; Wang et al. 2022a), and 97% for Lithium and 98% for Iron with phosphoric acid (Jiang et al. 2021; Hu et al. 2022)..

Selective leaching has been recently proposed for LIBs’ recycling (Kumar et al. 2022). It is based on leaching specific target elements from the black mass—Lithium leaching exceeded 97% (Jin et al. 2023; Wu et al. 2023), while other elements are extracted in consequent steps. Selective leaching involves mild organic acids, as citric (Kumar et al. 2020), formic (Mahandra and Ghahreman 2021), Methyl Sulfonic Acid (MSA) and p-Toluene Sulfonic Acid (TSA) (Prasad Yadav et al. 2020) in combination with an oxidizing agent, as hydrogen peroxide (Chen et al. 2018; Li et al. 2017; Tao et al. 2019; Zhou et al. 2023a), or sodium hypochlorite (K. Liu et al. 2023a, b; Liu et al. 2022; Tang et al. 2020)_._ Recent studies have proposed novel selective leaching processes based on sodium citrate solution, potentially increasing economic feasibility (Zhang et al. 2023a) or monosodium phosphate, reducing the generation of wastewater from recycling ( Zhou et al. 2023a). Moreover, due to shorter time required and lower concentration of leaching agents, compared to conventional hydrometallurgy, selective leaching can limit waste generation and reduce environmental impacts and economic costs (Kumar et al. 2022; K. Liu et al. 2023a, b). The environmental benefits of selective leaching could be enhanced by the use of organic acids,(Golmohammadzadeh et al. 2018; Zhou et al. 2023b), which are less persistent than inorganic ones and their application avoids the release of Cl_2_, NO_x_ and SO_3_ (Golmohammadzadeh et al. 2017; Meng et al. 2020). Furthermore, organic acids show highest selectivity towards Lithium leaching compared with inorganic acids, such as sulphuric or phosphoric acids (Gerold et al. 2023).

Citric acid was proposed as “green chemical” for LIBs’ hydrometallurgical recycling because it is soluble in water and naturally biodegradable (Li et al. 2010). However, despite organic acids are becoming increasingly common for metals’ leaching from Nickel Manganese Cobalt (NMC) and Lithium Cobalt Oxide (LCO) cathodes (Golmohammadzadeh et al. 2018; He et al. 2017; Kim et al. 2023; Zeng et al. 2015), they have been rarely applied to LFP recycling (Li et al. 2023; Wang et al. 2022b).

To the best of our knowledge, recycling EoL LFP cathodes via selective leaching with citric acid has not been yet extensively researched, and existing literature (Gerold et al. 2023; Li et al. 2019) focused on the efficiency of the process, overlooking the economic costs. When previous studies (Hu et al. 2024; P. Yadav et al. 2020; Yang et al. 2023) presented the economic analysis of recycling costs and potential profit from the recovery of Iron phosphate and Lithium carbonate, their results were controversial due to differences in functional units, currencies and costs parameters, e.g. reagents, energy, labour and general expenses. Moreover, previous studies often focused only on leaching, which is the initial step of hydrometallurgical recycling, (Jha et al. 2013; Jin et al. 2023) and when the sequential steps for the recovery of lithium carbonates is considered they report only the purity of the recovered carbonates and not the recovery efficiency (Wu et al. 2023).

The main objective and element of novelty of this study is the comparison of two closed-loop recycling routes applied to EoL LFP cathodes: selective leaching with citric acid at 25 °C and conventional leaching with sulfuric acid at 40 °C, both followed by recovery via chemical precipitation and solid-state synthesis of the recycled LFP phase, considering technical performance and economic analysis of the processes. Moreover, in this work the economic analysis has been performed both for a conventional hydrometallurgical process and for selective leaching on the same sample, considering the cost of reagents and energy consumption, during leaching and precipitation, with primary data measured during experimental activity.

Conventional leaching with sulfuric acid allowed to recover Li, Fe and P by multiple steps of chemical precipitation, whereas selective leaching with citric acid and hydrogen peroxide recovered Fe and P as residual material after leaching and used chemical precipitation only to recover Li, reducing the number of steps required for material recovery. The regeneration of recovered materials was carried out comparing two products: (i) Li, Fe and P precipitated after conventional leaching with sulfuric acid and (ii) Fe and P from the residues of selective leaching with citric acid and hydrogen peroxide and precipitated Li.The two routes of conventional leaching with sulfuric acid and selective leaching with citric acid and hydrogen peroxide have been assessed based on technical performances (leaching and recovery yields, and purity of recovered precursors to produce recycled LFP cathodes), and on associated costs referred to the treatment of 1 kg of EoL LFP cathodic powders.

## Materials and methods

### Materials and reagents

This study involved LFP cathodes provided by an Italian company, dismantled from EoL cells. The following reagents were used in the experimental tests: sulfuric acid (CAS: 7664–93-9, > 96% purity, Carlo Erba Reagents); citric acid (CAS: 77–92-9, > 99.5% purity, Sigma Aldrich); hydrogen peroxide (CAS: 7722–84-1, 30%v.v., Carlo Erba Reagents); sodium hydroxide (CAS: 1310–73-2, > 98% purity, Honeywell/Fluka); sodium carbonate (CAS: 497–19-8, > 99.8% purity, Sharlab); D( +)Glucose anhydrous (CAS: 50–99-7, > 97.5% purity, Carlo Erba Reagents).

## Analytical equipment

Before characterization, the samples were rinsed with deionized water and dried at 60 °C overnight in an ARGO LAB TCN 30 oven. A benchtop pH-meter (GEASS, PH8 + DHS) was used during leaching and chemical precipitation tests. The samples and recovered products have been characterized through: X-ray Fluorescence (XRF) spectroscopy (Rigaku, NEX-DE), X-ray Diffraction (XRD) spectroscopy (PANanalytical X’Pert) and Flame Atomic Absorption (FAA) spectroscopy (Schimadzu, GFA-EX7). Samples underwent microwave digestion before FAA spectroscopy in a microwave digestion system (MILESTONE, ETHOS UP), treating 500 mg with 50 mL of HNO_3_ (0.2 M) and HCl (0.8 M) at 230 °C for 25 min. XRF spectroscopy was used to measure the concentration of Al, Ca, Cl, Co, Cr, Cu, Fe, Ni, P, S, Si, V and Zn. Lithium was analysed through AAS. Iron was also analysed via AAS for consistency. It should be noticed that XRF directly analysed the powders, while AAS analyses were preceded by microwave acid digestion.

## Overview of the experimental approach

According to a previous study on pre-treatments (Bruno and Fiore 2024., in preparation), the LFP cathodes’ powders have been detached from the Aluminum current collectors via thermal treatment at 250 °C for 30 min in a Prederi ZE V220 muffle furnace, then ball milled for 5 min at 14 Hz in 50 mL Zirconia jars with two Zirconia beads (10 mm diameter) in a Retsch MM200 ball mill, and finally manually sieved to eliminate particles having dimensions above 1 mm.

**Fig. 1:**
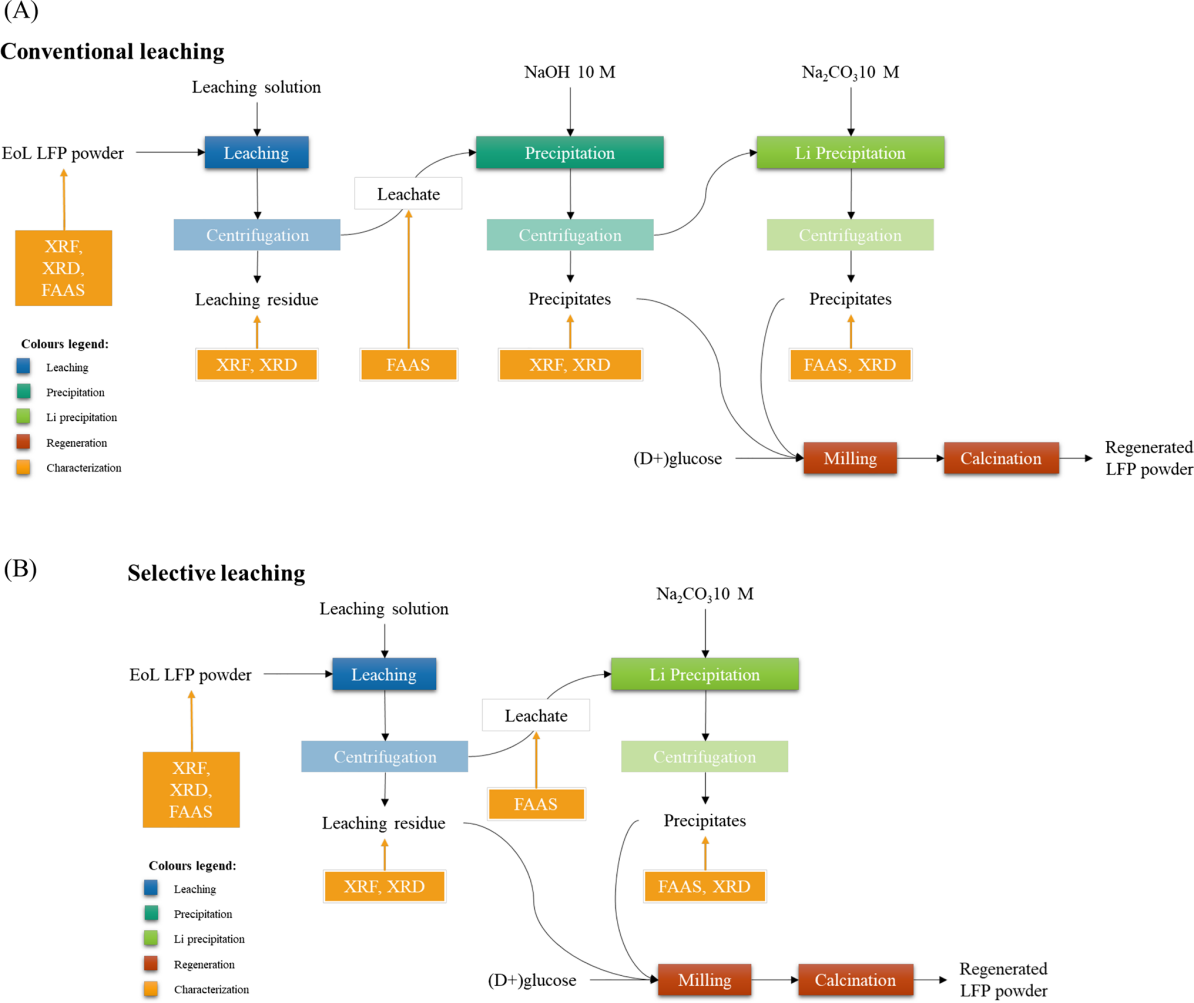
Outline of the experimental approach (**A**) with conventional leaching and (**B**) with selective leaching

The experimental activity applied in this study (Fig. 1) followed two recycling routes: conventional leaching and selective leaching. Conventional leaching involved 4 consequent phases: (i) leaching, (ii) Fe and P recovery via chemical precipitation with 10 M NaOH, (iii) Li recovery via chemical precipitation with 10 M Na_2_CO_3_, (iv) carbothermal reduction of LFP powders at 700 °C. While selective leaching involved 3 consequent phases: (i) leaching, (ii) Li recovery via chemical precipitation with 10 M Na_2_CO_3_, (iii) carbothermal reduction of LFP powders at 700 °C. All processes have been explored in triplicates. pH values adopted during Li, Fe and P recovery are detailed in the following.

Sulfuric and citric acids were compared as leaching agents for conventional leaching (Fig. 1A). Besides, citric acid in various conditions was combined with hydrogen peroxide in a selective leaching process (Fig. 1B). The experimental conditions applied (Table 1) are based on literature (Kumar et al. 2022; Qin et al. 2019; Sattar et al. 2019; Takahashi et al. 2020; Vieceli et al. 2021; Yue et al. 2018). Leaching tests have been carried out in a temperature-controlled Pyrex reactor placed on an AREX-6DIGITAL PRO heating magnetic stirrer and equipped with a VTF EVO digital thermoregulatory, both from VELP Scientifica. The solid residues have been recovered by a Hermle Labor Technik Z 206 A centrifuge, rinsed with deionized water, dried at 70 °C in an Argo Lab TCN 30 oven, and analyzed through XRF and XRD spectroscopy. The leachates have been filtered at 0.45 μm with GVS syringe filters and analyzed by FAA spectroscopy. pH of the leachates was increased by adding 10 M NaOH to precipitate Iron Phosphate (pH 2) and Iron Phosphate^.^8H_2_O (vivianite, pH 5.5), then recovered via centrifugation. Lithium was recovered as phosphate, by adding 10 M NaOH to the leachate up to pH 11, then as Li_2_CO_3_ by adding 10 M Na_2_CO_3_ at 95 °C for 2 h. The recovered powders have been combined according to the stoichiometric ratio Li:Fe:P = 1:1:1, adding 20%wt. D( +)glucose as carbon source, and ball milled in a Retsch MM400 mill for 3 h at 20 Hz. Finally, the recovered powders have been thermally treated at 700 °C for 4 h in a Carbolite MTF 12/38/400 tubular furnace under Argon to obtain recycled LFP.

**Table 1:** Leaching conditions (concentration of leaching agent and hydrogen peroxide, temperature, contact time, S/L: solid to liquid ratio) applied in this study

Leaching agent	Concentration (M)	Concentration H_2_O_2_ (%vv.)	Temperature (°C)	Time (h)	S/L ratio (g/L)	Reference
sulfuric acid	3	4	50	0.5	0.05	Sattar et al. 2019
sulfuric acid	3	0	70	1.5	10	Qin et al. 2019
sulfuric acid	2.25	0	80	0.5	100	Yue et al. 2018
sulfuric acid	2	0	-	1	20	Vieceli et al. 2021
sulfuric acid	1.2	0	25–50	4	0.2	Takahashi et al. 2020
citric acid	0.25	6	25	1.5	67	Kumar et al. 2022
sulfuric acid	1	0	40	2	50	This study (S_0_)
citric acid	1	0	25	1	70	This study (C_0_)
citric acid	1	6	25	1	70	This study (C_1_)
citric acid	0.5	6	25	1	70	This study (C_2_)
citric acid	0.25	6	25	1	70	This study (C_3_)

The overall efficiency of the investigated processes has been assessed via two performance indicators, e.g., leaching efficiency (η_leach_) and recovery efficiency (η_rec_):

$${\eta }_{leach}\left(\%\right)=\frac{{c}_{i}\left(mg/l\right)\bullet {V}_{i} (l)}{{m}_{i}(mg)}\bullet 100$$

$${\eta }_{rec}\left(\%\right)=\frac{\Sigma {m}_{prec}\left(mg\right)}{{m}_{i}(mg)}\bullet 100$$

where c_i_ is the concentration of Li, Fe and P in the leachate (mg/L), V_i_ is the leachate volume (L), m_i_ is the mass of Li, Fe and P in the initial sample (mg) and m_rec._ is the mass of Li, Fe and P in the recovered powders (mg). Moreover, the purity of the recovered compounds was investigated via chemical and XRD analyses.

## Economic preliminary analysis

The economic analysis of the investigated processes was based on 1 kg of LFP powder and accounted the costs of recycling (Table 2), considering the costs due to energy demand and reagents. This analysis should be considered purely preliminary and aimed at just comparing the economic aspects associated to the compared routes. The energy consumption of the lab equipment was measured with a PM10 Maxcio power meter. The analysis accounted the average European price of electricity for non-households consumers, equal to 0.1986 €/kWh (Eurostat 2023). The costs of reagents and deionized water have been retrieved from Ecoinvent database (Ecoinvent 2023).

**Table 2:** Costs accounted in the preliminary economic analysis

Costs	**Market value**	**m.u**
Energy	0.199	€/kWh
Deionized water	8.08·10^–5^	€/kg
Sulfuric acid	0.06	€/kg
Citric acid	0.78	€/kg
Hydrogen peroxide	0.47	€/kg
Sodium hydroxide	0.19	€/kg
Sodium carbonate	0.24	€/kg

## Results and discussion

### Samples’ characterization

**Fig. 2:**
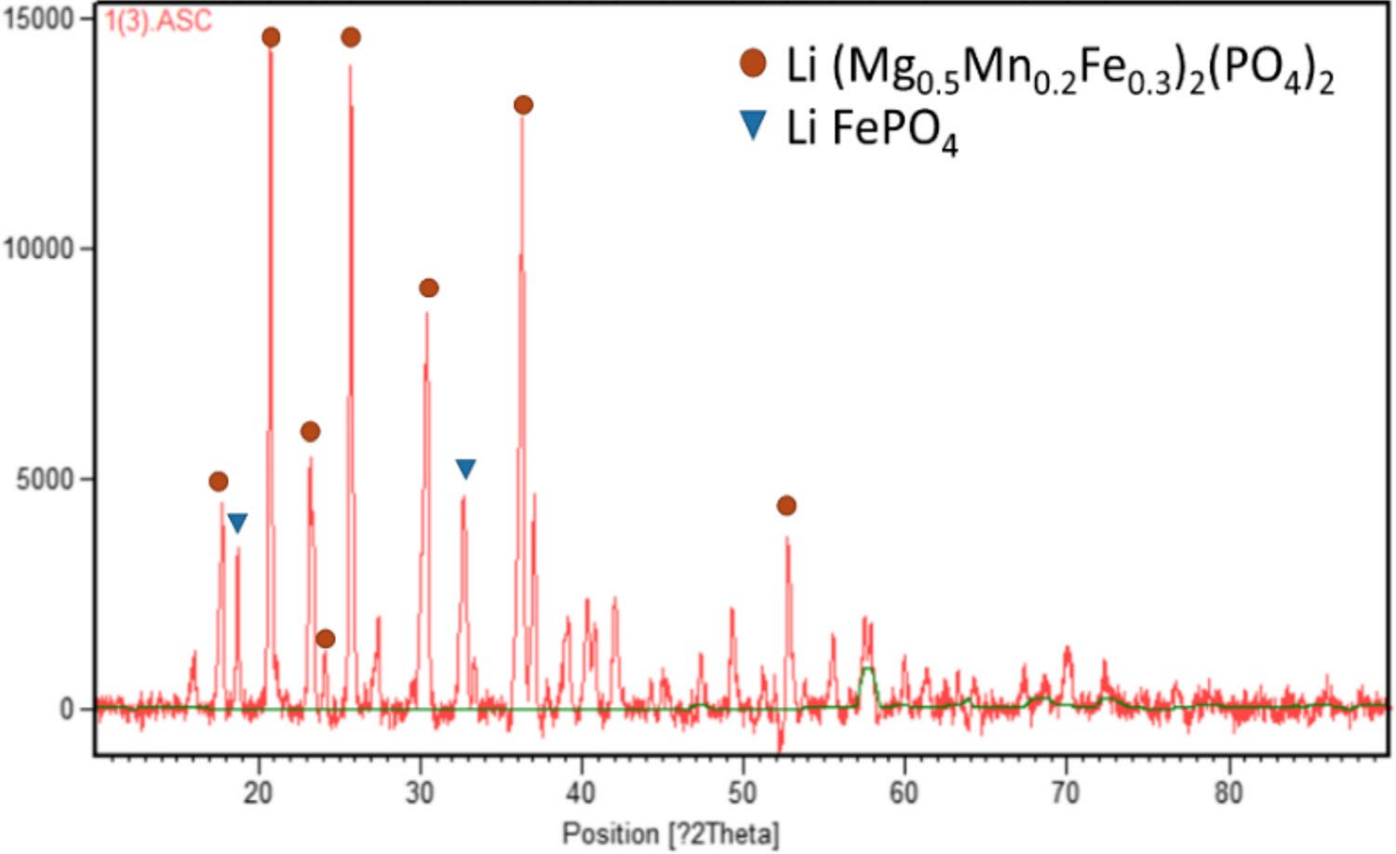
XRD spectra of EoL LFP cathodic powders

According to the characterization’s results (Fig. 2), the EoL LFP cathodic powders have been identified as Lithium Iron Phosphate and Li-Mg-Mn Iron Phosphate. Their chemical composition was: 1.6 ± 0.01%wt. Li, 29.64 ± 1.32%wt. Fe and 14.80 ± 0.4%wt. P, in agreement with literature (Gaines et al. 2018; Yagci et al. 2021; Yang et al. 2018; Zhang et al. 2022). Aluminium impurities from the current collector were 0.092 ± 0.06%-wt., proving the efficiency of the applied detachment process.

## Leaching processes

**Fig. 3:**
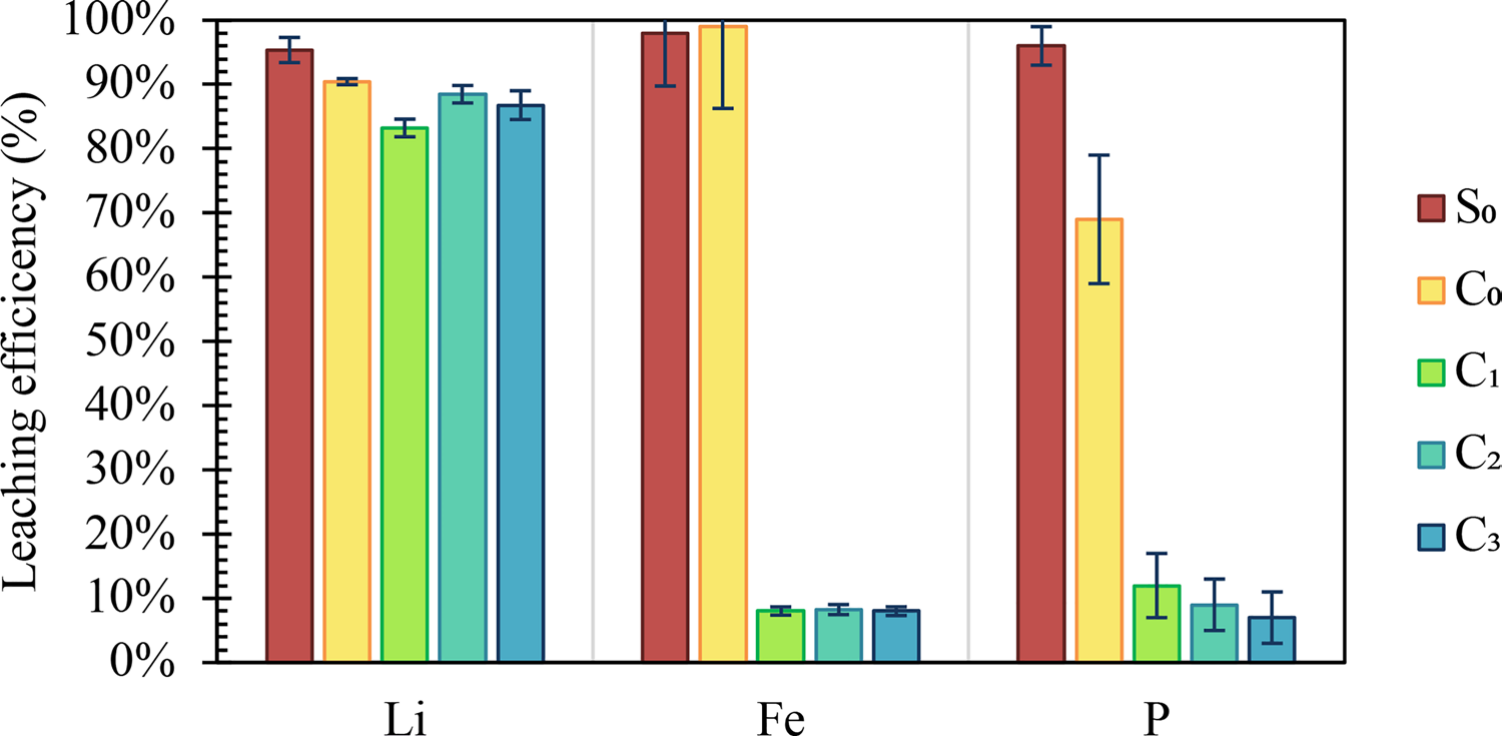
Leaching efficiency of Lithium, Iron and Phosphorous achieved in the performed tests (S_0_ = sulfuric acid 1 M, C_0_ = citric acid 1 M, C_1_ = citric acid 1 M + 6%v.v. H_2_O_2_, C_2_ = citric acid 0.5 M + 6% v.v. H_2_O_2_, C_3_ = citric acid 0.25 M + 6% v.v. H_2_O_2_)

The results of leaching tests (Fig. 3) were as follows. Sulfuric acid was the most effective leaching agent, extracting 95 ± 2% of Li, 98 ± 8% of Fe, and 96 ± 3% of P. Similar values have been reported by a previous study (Zheng et al. 2016), which leached 97% of Li and 98% of Fe with higher sulfuric acid concentration and longer contact time but lower solid-to-liquid ratio. Citric acid exhibited comparable leaching efficiency: 90 ± 0.6% of Li, 99 ± 13% of Fe, and 69 ± 10% of P. Aside from the fact that sulfuric acid is stronger than citric, the lower performance of citric acid, compared to sulfuric, may be due to the milder leaching conditions (25 °C applied for 1 h vs. 40 °C and 2 h).

The leaching mechanisms involved in the leaching process with sulfuric acid and citric acid are provided in Eq. 1 and Eq. 2. While the selective leaching mechanism, due to the presence of hydrogen peroxide which acts as an oxidant agent, is reported in Eq. 3.

1 $$2 LiFeP{O}_{4}+ {{\text{H}}}_{2}{{\text{SO}}}_{4}\to 2\mathrm{ FeS}{{\text{O}}}_{4}+{{\text{Li}}}_{2}{\text{SO}}4+2 {{\text{H}}}_{3}{{\text{PO}}}_{4}$$

2 $$3 {H}_{3}cit +3 LiFeP{O}_{4} \to F{e}_{3} (cit{)}_{2} + L{i}_{3}cit + 3 {H}_{3}P{O}_{4}$$

3 $$2 {H}_{3}cit+ 3 {H}_{2}{O}_{2}+ 6 LiFeP{O}_{4} \to 6 FeP{O}_{4} + 2 L{i}_{3}cit+6 {H}_{2}O$$

The selectivity of Li leaching is achieved by the effect of hydrogen peroxide, which oxidize Fe^2+^ into Fe^3+^, strenghtening the olivine structure of FePO_4_, as reported by previous studies (Niu et al. 2023). A similar result has been presented, without hydrogen peroxide, by using a stoichiometric amount of sulfuric acid, which however was selective towards Fe but still leached 20% of P (Tao et al. 2019).

Contrary to previous studies, which reported an increase of Li leaching efficiency due to the presence of hydrogen peroxide (Mahandra and Ghahreman 2021), it had no effect on Lithium leaching efficiciency. Specifically, the amount of Li leached with 1 M citric acid and 6%v.v. hydrogen peroxide (tests C_1_, C_2_ and C_3_) was 92 ± 2% of Li leached without hydrogen peroxide. On the other hand, it had a detrimental effect on the leaching efficiencies of Fe and P. This study additionally proved that, in presence of 6%v.v. hydrogen peroxide, Fe and P leaching efficiency was unaffected when citric acid concentration decreased, as follows. 83 ± 0.8% Li, 8 ± 1% Fe, and 12 ± 5% P were leached with 1 M citric acid; 88 ± 1% Li, 8 ± 1% Fe, and 9 ± 4% P with 0.5 M citric acid; and 87 ± 2% Li, 8 ± 1% Fe, and 7 ± 4% P with 0.25 M citric acid.

## Iron and phosphorous recovery

**Fig. 4:**
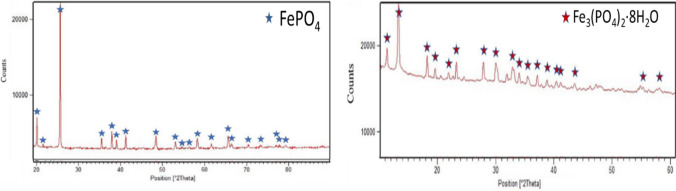
XRD spectra of the recovered powders obtained from sulfuric acid leaching at (**A**) pH 2 after thermal treatment at 700 °C, and at (**B**) pH 5.5

Following conventional leaching with sulfuric acid, 69 ± 15% Fe and 70 ± 18% P have been recovered at room temperature from the leachates by adding NaOH to precipitate FePO_4_ at pH 2 and vivianite FePO_4_^.^H_2_O at pH 5.5 (Fig. 4). In particular, FePO_4_ obtained at pH 2 didn’t display a crystalline structure, thus it was treated at 700 °C for 3 h (Fig. 4A). The recovery of Fe and P via chemical precipitation at room temperature required less than 20 min, with comparable shares of Iron Phosphate obtained at pH 2.2 (53 ± 8%wt. of total precipitates) and Vivianite at pH 5.5 (41 ± 10%wt. of total precipitates) (Fig. 4B).

**Fig. 5:**
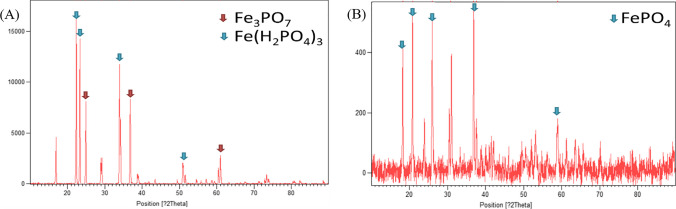
XRD spectra of the recovered powders obtained from citric acid leaching at (**A**) pH 2 after thermal treatment at 700 °C, and at (**B**) pH 5.5

Chemical precipitation with NaOH was also applied to recover Fe and P after conventional leaching with citric acid and achieved the recovery of 82 ± 6% for Fe and 74 ± 8% for P, as Iron phosphate and hydroxyphosphate (Fig. 5).

Following conventional sulfuric acid leaching, three precipitation processes were carried out, followed by six centrifugation steps, since it was required to repeat the centrifugation to guarantee better separation. The leachate with citric acid instead required two precipitation and four centrifugation steps.

Whereas, selective leaching allowed to recover Fe and P as FePO_4_ in the solid residues after leaching and the leachate required only one precipitation and centrifugation step to remove Fe impurities (2 ± 1% wt.). Indeed, selective leaching presented the following recovery efficiency: 92 ± 1% of Fe and 88 ± 5% of P with citric acid 1 M and 6%vv. hydrogen peroxide, 92 ± 1% of Fe and 91 ± 4% of P with citric acid 0.5 M and 6%vv. hydrogen peroxide, and 92 ± 1% of Fe and 93 ± 4% of P with citric acid 0.25 M and 6%vv. hydrogen peroxide.

## Lithium recovery

**Fig. 6:**
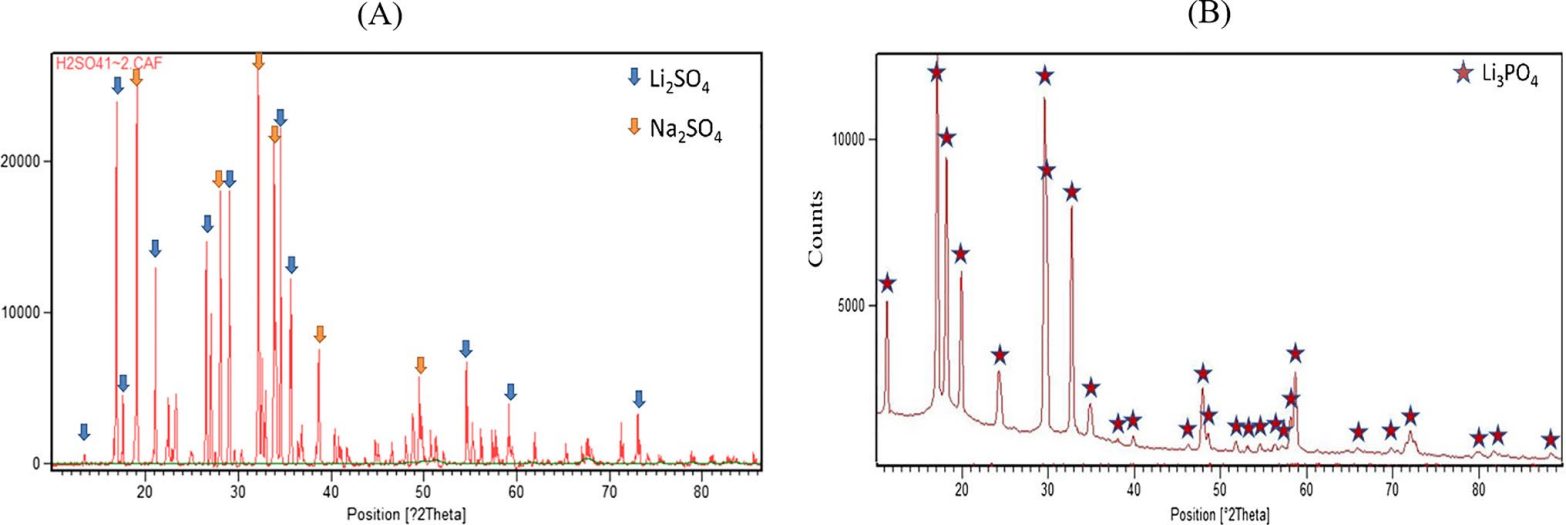
XRD spectra of precipitates obtained at pH 12 from (**A**) conventional sulfuric acid leachates and (**B**) selective 0.25 M citric acid and hydrogen peroxide leachates

Lithium recovery was completed comparing two leaching processes: conventional leaching with sulfuric acid and selective leaching with citric acid and hydrogen peroxide. Lithium recovery happened at 95 °C, in two phases. Firstly, pH of leachates was increased at 12 with 10 M NaOH, then a stoichiometric amount of Na_2_CO_3_ was added to precipitate Li as carbonate. Depending on the leaching process, different precipitates have been obtained in the first phase (Fig. 6): lithium sulphate (98 ± 12%wt. of recovered Lithium) with conventional sulfuric acid, and lithium phosphate (66 ± 7%wt. of recovered Lithium) with citric acid and hydrogen peroxide.Lithium recovery as Li_3_PO_4_ from LFP cathodes was previously reported by literature (Mahandra and Ghahreman 2021) and.the precipitation of Li phosphate alongside Li carbonate is attributed to the fact that the solubility constant value is lower for Li_3_PO_4_ (2.37·10^–11^) than for Li_2_CO_3_ (8.15·10^–4^) (Lide 2004).At 95°C, Na_2_CO_3_ was added to the leachate to precipitate the residual Lithium as carbonate, achieving a total recovery of 21 ± 2% from sulfuric acid route (98 ± 12% as Li_2_SO_4_ and 2 ± 0.7% as Li_2_CO_3_) and 29 ± 5% from citric acid and hydrogen peroxide route (66 ± 7% % as Li_3_PO_4_ and 34 ± 5% as Li_2_CO_3_). In this study, Lithium recovery efficiency from selective leaching was lower compared to literature considering selective leaching (Kumar et al. 2020) and conventional leaching (Dolotko et al. 2020), which reported values between 70 and 85%wt. A possible explanation of the poor performances of Li recovery achieved in this study may be associated with the relatively low masses involved in this study (4 g of EoL LFP cathodic powders producing 0.86 g of Lithium-rich precipitates from conventional leaching with sulfuric acid and 1.16 g from selective leaching with citric acid and hydrogen peroxide), which were probably affected by material losses during the overall leaching and recovery processes.

The recovery of lithium was carried out, after conventional and selective leaching, with two precipitations steps (after reaching pH 12, and after adding Na_2_CO_3_ at 95 °C) and two centrifugation steps to separate the precipitates.

## Purity of recovered compounds

**Fig. 7:**
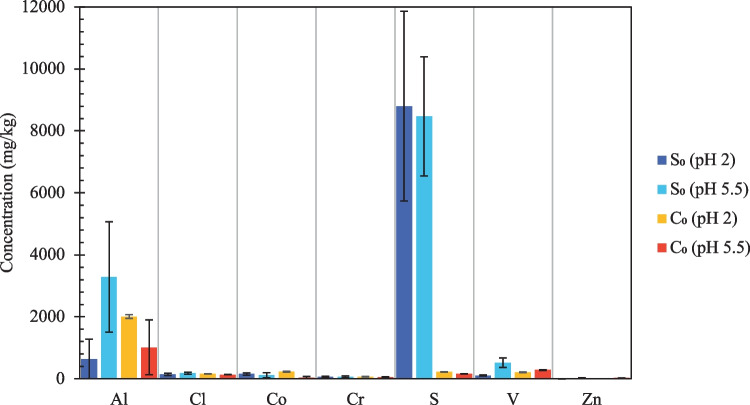
Concentration of impurities in the precipitates from conventional leaching (S_0_ sulfuric acid 1 M; C_0_ citric acid 1 M)

**Fig. 8:**
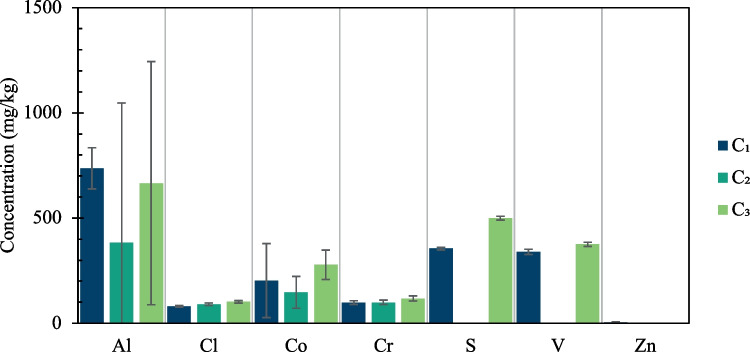
Concentration of impurities in the solid residues from selective leaching (C_1_ = citric acid 1 M + 6%v.v. H_2_O_2_, C_2_ = citric acid 0.5 M + 6% v.v. H_2_O_2_, C_3_ = citric acid 0.25 M + 6% v.v. H_2_O_2_)

The performance of the recycling processes was evaluated considering also the concentration of impurities in the Fe- and P-rich powders, through XRF spectroscopy to determine the concentration of other elements in the products of precipitation from conventional leaching (Fig. 7) and the residues from selective leaching (Fig. 8). The concentration of Cu, Ni and Si was below the detectable limit of XRF spectroscopy.

The most prevalent contaminant in the Fe- and P-rich powders recovered from sulfuric acid route was S, which was 8798 ± 3061 mg/kg in Iron Phosphate recovered at pH 2 and 8468 ± 1925 mg/kg in Vivianite recovered at pH 5.5. Other contaminants detected in same powders were Co, mostly leached at pH 2 (153 ± 37 m/kg of precipitate), while Vivianite contained Al (3287 ± 1777mg/kg), V (520 ± 155 mg/kg) and Cl (181 ± 31 mg/kg). In overall, the precipitated fraction from conventional leaching with sulfuric acid presented the following contaminations: 1681 ± 1063 mg/kg of Al, 149 ± 31 mg/kg of Cl, 128 ± 51 mg/kg of Co, 8104 ± 2403 mg/kg of S and 270 ± 71 mg/kg of V.

Powders recovered via conventional leaching with citric acid displayed contaminations of Al (2010 ± 62 mg/kg), V (219 ± 15 mg/kg) and Cl (160 ± 7 mg/kg) at pH 2 and of Al (1016 ± 881mg/kg), V (158 ± 13 mg/kg) and Cl (136 ± 5 mg/kg) at pH 5.5. The totality of contamination in the precipitates from conventional leaching with citric acid were: 1602 ± 381 mg/kg of Al, 149 ± 7 mg/kg of Cl, 155 ± 24 mg/kg of Co, 193 ± 7 mg/kg of S and 234 ± 14 mg/kg of V.

The residues from selective leaching presented lower concentration of Al, S, Zn and Cl compared with the precipitates from conventional leaching. In particular the contaminations in the leaching residues after selective leaching were: 736 ± 97 mg/kg of Al, 80 ± 4 mg/kg of Cl, 202 ± 176 mg/kg of Co, 354 ± 7 mg/kg of S and 339 ± 12 mg/kg of V with citric acid 1M and hydrogen peroxide 383 ± 663 mg/kg of Al, 91 ± 5 mg/kg of Cl, 146 ± 75 mg/kg of Co, 331 ± 19 mg/kg of S and 328 ± 2 mg/kg of V with citric acid 0.5M and hydrogen peroxide and 665 ± 557 mg/kg of Al, 101 ± 6 mg/kg of Cl, 277 ± 69 mg/kg of Co, 499 ± 10 mg/kg of S and 357 ± 9 mg/kg of V with citric acid 0.25M and hydrogen peroxide.

The Lithium-rich powders from conventional leaching with sulfuric acid contained: 978 ± 853 mg/kg of Al, 417 ± 17 mg/kg of Cl, 51,900 ± 2165 mg/kg of S, detected as Na_2_SO_4_ (Fig. 6A). The concentration of Co, Cr and V were below detection limits. Whereas the products of lithium recovery from selective leaching with citric acid and hydrogen peroxide presented the following contaminations: 7523 ± 187 mg/kg of Al, 236 ± 71 mg/kg of Cl, 335 ± 19 mg/kg of S and 236 ± 71 mg/kg of V.

## Carbothermal reduction of recovered LFP powders

**Fig. 9:**
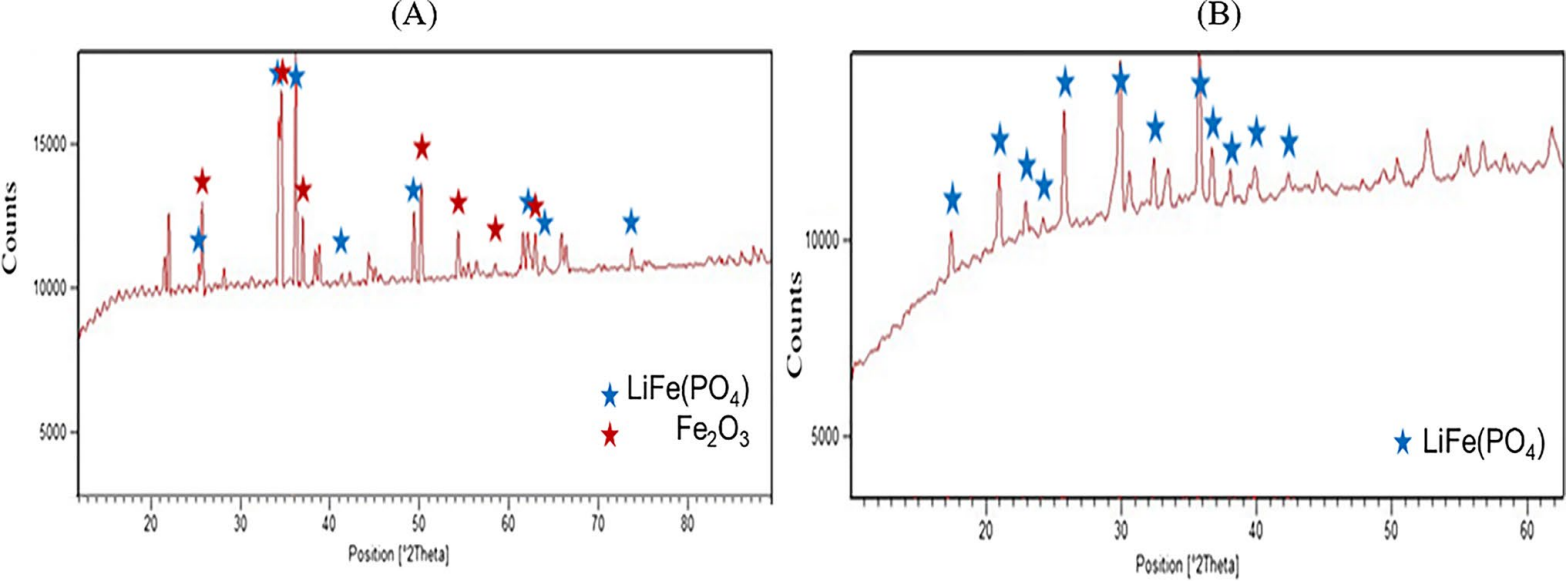
XRD spectra of recycled LFP powders deriving from (**A**) sulfuric acid route and from (**B**) selective citric acid route

The recovered powders deriving from sulfuric acid and 0.25 M citric acid routes underwent carbothermal reduction to obtain precursors for recycled LFP cathodes. Lithium Iron Phosphate Olivine crystalline structure was detected in the XRD spectra of both materials (Fig. 9), with iron oxides impurities in LFP powders deriving from sulfuric acid route. Since XRF spectroscopy was not able to differentiate the amount of iron present in form of oxides and in form of lithium iron phosphate, it was not possible to quantify the amount of iron oxides impurities.

## Economic analysis

Conventional sulfuric acid leaching required one leaching step at 40 °C, followed by three precipitation and six centrifugation steps to recover the precipitates and two precipitation steps (one at pH 12 and room temperature and one at 95 °C) to recover the lithium-rich precipitates; the precipitates were eventually regenerated by carbothermal reduction. Instead, selective leaching required one leaching step at 25 °C, one precipitation and two centrifugation steps to remove residual Fe impurities, and two precipitation steps (one at pH 12 and room temperature and one at 95 °C) to recover Lithium-rich precipitates; the leaching residues and precipitated Lithium were regenerated by carbothermal reduction.

**Fig. 10:**
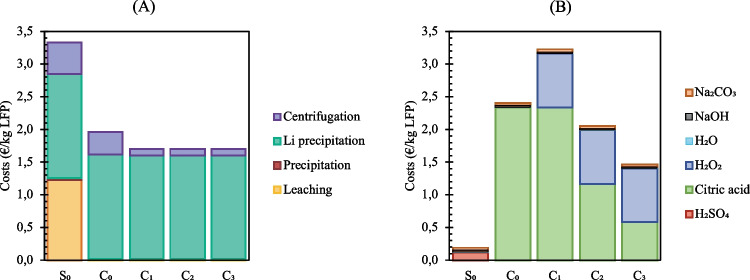
Costs associated with (A) energy demand and (B) reagents’ consumption (S_0_ sulfuric acid 1 M; C_0_ citric acid 1 M; C_1_ citric acid 1 M + 6% H_2_O_2_; C_2_ citric acid 0.5 M + 6% H_2_O_2_; C_3_ citric acid 0.25 M + 6% H_2_O_2_)

The overall operative costs of the compared recycling routes (Fig. 10) were 3.52 € per kg of recycled LFP for 1 M sulfuric acid leaching, and 4.37 €/kg for 1 M citric acid leaching without hydrogen peroxide. Lowering citric acid concentration and adding hydrogen peroxide reduced the costs of selective leaching: from 4.93 €/kg of recycled LFP for 1 M, to 3.76 €/kg for 0.5 M and 3.17 €/kg for 0.25 M. The most expensive reagents were hydrogen peroxide (0.82 €/kg of recycled LFP) and citric acid (2.34 €/kg of recycled LFP for 1 M, 1.17 €/kg for 0.5 M and 0.59 €/kg for 0.25 M). Due to the increased temperatures necessary to precipitate Li_2_CO_3_, lithium recovery displayed the highest energy demand (1.59 €/kg).

In overall, sulfuric acid route’s costs were mostly (94%) related to energy demand, while chemical reagents H_2_SO_4_, NaOH and Na_2_CO_3_ accounted for 0.20 € per kg of recycled LFP. Costs of 1 M Citric acid route were balanced between chemicals (55%) and energy demand (45%). When hydrogen peroxide was added and citric acid concentration decreased to 0.25 M, the costs’ partition changed, e.g., 46% was ascribable to chemicals and 54% to energy demand.

Previous studies considered the economic costs and potential profit from recycling LFP batteries (Table 3), their results have been converted into a comparable unit of measurement (€/kg LFP). According to literature, the average cost of LFP recycling corresponds to 15.5 ± 22.4 €/kg. The substantial discrepancy in this result is credited to the fact that each study considered various cost factors (raw materials, reagents, energy consumption, labour, equipment and overhead expenses). and process steps. In particular all the considered studies considered the cost of reagentes, almost 80% considered the costs of energy consumption, 50% considered the price of spent LFP batteries as raw material for the process, below 30% considered additional costs, such as labour cost, equipment maintenance, plant overhead and general expenses and only 8% of them considered the expenses due to waste management. Moreover, less than 38% of the studies considered the costs associated with pre-treatment or final regeneration of the recovered products and only 17% take into account both steps. Our study exclusively concerned the cost of reagents and energy consumption, during leaching and precipitation, since these metrics were collected as primary data during experimental activity.

**Table 3:** Summary of economic analysis results from literature overview on LFP recycling

Functional unit	Process	Costs	Income	Profit	Costs (€)	Considered costs										Process steps						Reference
						Raw materials	Reagents	Energy	Labour	Equipment	Waste management	Fixed charges	Plant overhead	General Expenses	Pre-treatment	Mechanochemical activation		Leaching	Recovery	Electrolysis	Regeneration	
1 kg of LFP (input)	Selective leaching	15.98 $	17.79 $	1.82 $	14.66	**x**	**x**	**x**	**x**						**x**			**x**	**x**		**x**	( Zhou et al. 2023a)
	Selective leaching	8.27 $	9.98 $	1.71 $	7.59	**x**	**x**	**x**	**x**									**x**	**x**			( Zhou et al. 2023b)
	Mechanochemical with Na-citrate	32.8 $	44.7 $	11.9 $	30.09	**x**	**x**	**x**										**x**	**x**			(Zhang et al. 2023b)
1 kg of cell (input)	Electrolysis			25.8 $			**x**	**x**												**x**		(Zhao et al., 2024)
1 kg of LFP battery (input)	Leaching	1.65 $	5.97 $	4.32 $	7.0		**x**					**x**	**x**	**x**				**x**	**x**		**x**	(Jin et al. 2023)
	Leaching with H_2_SO_4_ and H_2_O_2_	2.86 $	5.85 $	2.99 $	12.1		**x**	**x**	**x**	**x**		**x**	**x**	**x**	**x**			**x**	**x**			(Xu et al. 2023)
	Leaching with H_2_SO_4_ and H_2_O_2_	15.81 $	19.84 $	4.03 $	66.8	**x**	**x**	**x**										**x**	**x**			(Yang et al. 2023)
	H_2_O_2_ method	1.25 $	8.98 $	7.73 $	5.3		**x**					**x**	**x**	**x**				**x**	**x**		**x**	(Jin et al. 2023)
	Leaching with CO_2_ and H_2_O_2_	2.38 $	6.13 $	3.75 $	10.1		**x**	**x**	**x**	**x**		**x**	**x**	**x**	**x**			**x**	**x**			(Xu et al. 2023)
	Leaching with CO_2_ and O_2_	2.09 $	6.13 $	4.04 $	8.8		**x**	**x**	**x**	**x**		**x**	**x**	**x**	**x**			**x**	**x**			(Xu et al. 2023)
	Self-catalytic air pressure leaching	1.11 $	9.09 $	7.98 $	4.7		**x**					**x**	**x**	**x**				**x**	**x**		**x**	(Jin et al. 2023)
	Mechanochemical	1.85 $	6.62 $	4.768	7.8	**x**	**x**	**x**							**x**		**x**	**x**	**x**		**x**	(Liu et al. 2023a, b)
1 t of LFP battery (input)	Leaching with H_2_SO_4_	1,418 $	2,716 $	1,297 $	5.04	**x**	**x**	**x**										**x**	**x**			(Song et al. 2021)
	Selective leaching	26,499 $	40,586 $	7,469 $	94.23	**x**	**x**								**x**			**x**			**x**	(Hu et al. 2022)
	Selective leaching	35,700 ¥	54,520 ¥	18,820 ¥	0.86	**x**	**x**								**x**			**x**	**x**			(Song et al. 2023)
	Selective leaching and precipitation	1962.4 $	1242.4 $	719.6 $	6.98	**x**	**x**	**x**										**x**	**x**		**x**	(Hu et al. 2024)
	Selective air oxidation leaching	2,051 $	4,081 $	2,030 $	7.29	**x**	**x**	**x**	**x**	**x**					**x**			**x**	**x**			(Jin et al. 2022)
	Mechanochemical with FeCl_3_	1,580 $	6,539 $	4,959 $	5.62	**x**	**x**	**x**									**x**	**x**	**x**			(Wu et al. 2023)
	Electrolysis	2225 $	9148 $	6923 $	7.91	**x**	**x**	**x**												**x**		(Yang et al. 2024)
1 kg of recovered	Traditional leaching	18.60 $			17.06		**x**	**x**														(Du et al. 2024)
	Leaching with citric acid and MSA	4.35 $			3.99		**x**	**x**										**x**				(Kim et al. 2023)
	Selective pressure leaching with H_2_SO_4_	17.36 $			15.93		**x**	**x**														(Du et al. 2024)
1 kg of FePO_4_ leaching residues (input)	Regeneration of battery-grade FePO_4_ with H_3_PO_4_ ultrasonic centrifugation	1.25 $	1.80 $	0.55 $	1.15		**x**	**x**			**x**							**x**	**x**		**x**	(Han et al. 2023)
100 kWh	Leaching with methanesulfonic acid (MSA), p-toluenesulfonic acid (Ts	970 $	1,129 $	159 $			**x**	**x**	**x**	**x**	**x**			**x**	**x**			**x**	**x**		**x**	(Prasad Yadav et al. 2020)

Therefore, additional expenses that full scale recycling facility have, such as capital investment or operative expenses, e.g. labour cost, equipment maintenance and waste management, were not considered. This limitation prohibits to assess the economic feasibility of the process. Nonetheless, the direct comparison between operative costs of conventional and selective leaching processes allows to quantify the economic benefit of selective leaching towards conventional processes, that required higher process temperature and additional recovery steps.

## Conclusion

Despite hydrometallurgical recycling of lithium-ion batteries is widely applied at full-scale and intensively researched, current full-scale technologies are not economically profitable for Lithium Iron Phosphate batteries. This study had the main goal of comparing two closed-loop recycling routes applied to EoL LFP cathodic powders based on conventional leaching with sulfuric acid at 40 °C and on selective leaching with citric acid at 25 °C to recover and synthesize LFP precursors via chemical precipitation and solid-state synthesis of the recycled LFP phase. The comparison involved two objectives: (i) the technical performances of the two routes (yield of leaching and recovery, purity of the recovered powders), and (ii) a preliminary economic analysis, based on experimental data achieved from the study and compared to literature data, referred to 1 kg of end-of-life LFP cathodic material treated. In details, conventional leaching was performed with sulfuric acid and with citric acid, while selective Li leaching was performed with citric acid and hydrogen peroxide. Leaching efficiency of conventional leaching with citric acid (90 ± 0.6% for Li, 99 ± 13% for Fe, 69 ± 10% for P) was comparable with sulfuric acid (95 ± 2% for Li, 98 ± 8% for Fe, 96 ± 3% for P) considering that citric acid processes happened at 25 °C (instead of 40) and involved shorter contact time, higher solid to liquid ratio. Selective leaching, with hydrogen peroxide and lower citric acid concentration (from 1 to 0.25 M) showed comparable Li leaching efficiency (87 ± 2%) and allowed to recover 92 ± 1% of Fe and 93 ± 4% of P in the leaching residues. The route based on conventional leaching with sulfuric acid, was followed by chemical precipitation and recovered 69 ± 15% of Fe and 70 ± 18% of P, with contaminations of Al (1681 ± 1063 mg/kg), Cl (149 ± 31 mg/kg), Co (128 ± 51 mg/kg), S (8104 ± 2403 mg/kg) and V (270 ± 71 mg/kg). Whereas selective leaching with 0.25 M citric acid and 6%vv. hydrogen peroxide allowed to recover 92 ± 1% of Fe and 93 ± 4% of P in the leaching residues with the following contaminations: 665 ± 557 mg/kg of Al, 101 ± 6 mg/kg of Cl, 277 ± 69 mg/kg of Co, 499 ± 10 mg/kg of S and 357 ± 9 mg/kg of V. In total, the route based on conventional leaching with 1M sulfuric acid allowed to recover 21 ± 2% of Li, 69 ± 15% of Fe and 70 ± 18% of P. Objective (i) of the study—i.e. comparison of the technical performances of the two routes—has been achieved, as the performance of selective leaching with 0.25 M citric acid and hydrogen peroxide were better, compared to conventional leaching: 29 ± 5% of Li, 92 ± 1% of Fe and 93 ± 4% of P.

Objective (ii) of the study—i.e. preliminary economic assessment of the two routes—has been also achieved, as follows. The preliminary economic assessment revealed that 0.25 M citric acid route was cheaper (3.17 €/kg) than the one based on sulfuric acid (3.52 €/kg), because of the lower energy demand and fewer process phases. In conclusion, from the point of view of a general perspective, this study proved that selective leaching with citric acid and hydrogen peroxide, compared to sulfuric acid in the recycling of EoL LFP cathodic powders, can be considered a promising recycling route for Li, Fe and P and is worth of further research to improve Lithium recovery.

## References

[CR1] Bruno M, Fiore S (2024) Low cost and environmentally friendly physic-mechanical pre-treatments to recycle lithium iron phosphate batteries. J Environ Chem Eng 12:112106. 10.1016/j.jece.2024.112106

[CR2] Chan KH, Anawati J, Malik M, Azimi G (2021) Closed-loop recycling of lithium, cobalt, nickel, and manganese from waste lithium-ion batteries of electric vehicles. ACS Sustain Chem Eng 9:4398–4410. 10.1021/acssuschemeng.0c06869

[CR3] Chen X, Cao L, Kang D, Li J, Zhou T, Ma H (2018) Recovery of valuable metals from mixed types of spent lithium ion batteries. Part II: Selective extraction of lithium. Waste Manag 80:198–210. 10.1016/j.wasman.2018.09.013 30455000 10.1016/j.wasman.2018.09.013

[CR4] Du, J., Qing, J., Fang, K., Zhang, G., Cao, Z.(2024). The priority leaching of lithium from spent LiFePO 4 cathode without the oxidization. Resour Conserv Recycl 202, 107374. 10.1016/j.resconrec.2023.107374

[CR5] Du J, Qing J, Fang K, Zhang G, Cao Z (2024) The priority leaching of lithium from spent LiFePO 4 cathode without the oxidization. Resour Conserv Recycl 202:107374

[CR6] Ecoinvent, 2023. ecoinvent v3.8 - ecoinvent [WWW Document]. URL https://ecoinvent.org/the-ecoinvent-database/data-releases/ecoinvent-3-8/ Accessed 3 Aug 2023

[CR7] European Commission (2020) Green Deal: Sustainable batteries for a circular and climate neutral economy. https://commission.europa.eu/strategy-and-policy/priorities-2019-2024/european-green-deal_en. Accessed 10 Oct 2023

[CR8] Eurostat (2023) Electricity prices for non-household consumers - bi-annual data (from 2007 onwards) [WWW Document]. URL https://ec.europa.eu/eurostat/databrowser/view/nrg_pc_205/default/table?lang=en. Accessed 15 Sept 2023

[CR9] Farjana SH, Huda N, Parvez Mahmud MA, Saidur R (2019) A review on the impact of mining and mineral processing industries through life cycle assessment. J Clean Prod 231:1200–1217. 10.1016/j.jclepro.2019.05.264

[CR10] Fu X, Beatty DN, Gaustad GG, Ceder G, Roth R, Kirchain RE, Bustamante M, Babbitt C, Olivetti EA (2020) Perspectives on cobalt supply through 2030 in the face of changing demand. Environ Sci Technol 54:2985–2993. 10.1021/acs.est.9b04975 32072813 10.1021/acs.est.9b04975

[CR11] Gaines L, Richa K, Spangenberger J (2018) Key issues for Li-ion battery recycling. MRS Energy Sustain 5. 10.1557/mre.2018.13

[CR12] Gerold E, Lerchbammer R, Strnad C, Antrekowitsch H (2023) Towards a sustainable approach using mineral or carboxylic acid to recover lithium from lithium iron phosphate batteries. Hydrometallurgy 222:106187. 10.1016/j.hydromet.2023.106187

[CR13] Golmohammadzadeh R, Faraji F, Rashchi F (2018) Recovery of lithium and cobalt from spent lithium ion batteries (LIBs) using organic acids as leaching reagents: a review. Resour Conserv Recycl 136:418–435. 10.1016/j.resconrec.2018.04.024

[CR14] Golmohammadzadeh R, Rashchi F, Vahidi E (2017) Recovery of lithium and cobalt from spent lithium-ion batteries using organic acids: process optimization and kinetic aspects. Waste Manag 64:244–254. 10.1016/j.wasman.2017.03.037 28365275 10.1016/j.wasman.2017.03.037

[CR15] Han F, Fang D, Feng Y, Fan Y, Wei Y, Liu Y, Qu L (2023) The recovery of high purity iron phosphate from the spent lithium extraction slag by a simple phosphoric acid pickling. Sep Purif Technol 323:124358. 10.1016/j.seppur.2023.124358

[CR16] He LP, Sun SY, Mu YY, Song XF, Yu JG (2017) Recovery of lithium, nickel, cobalt, and manganese from spent lithium-ion batteries using l -tartaric acid as a leachant. ACS Sustain Chem Eng 5:714–721. 10.1021/acssuschemeng.6b02056

[CR17] Hu, G., Gong, Y., Peng, Z., Du, K., Huang, M., Wu, J., Guan, D., Zeng, J., Zhang, B., Cao, Y. (2022) Direct Recycling Strategy for Spent Lithium Iron Phosphate Powder: An Efficient and Wastewater-Free Process. ACS Sustain Chem Eng 10.1021/acssuschemeng.2c03520

[CR18] Hu G, Huang K, Du K, Peng Z, Cao Y (2024) Efficient recovery and regeneration of FePO4 from lithium extraction slag : towards sustainable LiFePO4 battery recycling. J Clean Prod 434:140091. 10.1016/j.jclepro.2023.140091

[CR19] IEA (2023) Global EV Outlook 2023. www.iea.org Accessed 10 Oct 2023

[CR20] N Jantunen S Virolainen T Sainio 2022 Direct production of Ni–Co–Mn mixtures for cathode precursors from cobalt-rich lithium-ion battery leachates by solvent extraction Metals (Basel). 12 10.3390/met12091445

[CR21] Jha MK, Kumari A, Jha AK, Kumar V, Hait J, Pandey BD (2013) Recovery of lithium and cobalt from waste lithium ion batteries of mobile phone. Waste Manag 33:1890–1897. 10.1016/j.wasman.2013.05.008 23773705 10.1016/j.wasman.2013.05.008

[CR22] Jiang Y, Chen X, Yan S, Li S, Zhou T (2021) Pursuing green and efficient process towards recycling of different metals from spent lithium-ion batteries through Ferro-chemistry. Chem Eng J 426:131637. 10.1016/j.cej.2021.131637

[CR23] Jin H, Zhang J, Wang D, Jing Q, Chen Y, Wang C (2022) Facile and efficient recovery of lithium from spent LiFePO4 batteries via air oxidation–water leaching at room temperature. Green Chem 24:152–162. 10.1039/d1gc03333f

[CR24] Jin H, Zhang J, Yang C, Ma L, Chen Y, Wang C (2023) Selective recovery of lithium from spent LiFePO4 battery via a self-catalytic air oxidation method. Chem Eng J 460:141805. 10.1016/j.cej.2023.141805

[CR25] Kim JY, Wu J, Woo E, Yoo K, Kim J, Jae J, Jai L, Byeon W, Ahn J, Lee J (2023) Recycling for recovery of critical metals from ­ LiCoO2 cathode material through methanesulfonic acid - citric acid organic leaching system. mining. Metall Explor 40:1455–1467. 10.1007/s42461-023-00837-8

[CR26] Kumar J, Neiber RR, Park J, Ali Soomro R, Greene GW, Ali Mazari S, Young Seo H, Hong Lee J, Shon M, Wook Chang D, Yong Cho K (2022) Recent progress in sustainable recycling of LiFePO4-type lithium-ion batteries: Strategies for highly selective lithium recovery. Chem Eng J 431:133993. 10.1016/j.cej.2021.133993

[CR27] Kumar J, Shen X, Li B, Liu H, Zhao J (2020) Selective recovery of Li and FePO4 from spent LiFePO4 cathode scraps by organic acids and the properties of the regenerated LiFePO4. Waste Manag 113:32–40. 10.1016/j.wasman.2020.05.046 32505109 10.1016/j.wasman.2020.05.046

[CR28] Li H, Xing S, Liu Y, Li F, Guo H, Kuang G (2017) recovery of lithium, iron, and phosphorus from spent LiFePO4 batteries using stoichiometric sulfuric acid leaching system. ACS Sustain Chem Eng 5:8017–8024. 10.1021/acssuschemeng.7b01594

[CR29] Li L, Bian Y, Zhang X, Yao Y, Xue Q, Fan E, Wu F, Chen R (2019) A green and effective room-temperature recycling process of LiFePO4 cathode materials for lithium-ion batteries. Waste Manag 85:437–444. 10.1016/j.wasman.2019.01.012 30803599 10.1016/j.wasman.2019.01.012

[CR30] Li L, Ge J, Wu F, Chen R, Chen S, Wu B (2010) Recovery of cobalt and lithium from spent lithium ion batteries using organic citric acid as leachant. J Hazard Mater 176:288–293. 10.1016/j.jhazmat.2009.11.026 19954882 10.1016/j.jhazmat.2009.11.026

[CR31] Li P, Luo S, Zhang L, Wang Y, Zhang H, Wang J, Yan S, Hou P, Wang Q, Zhang Y, Liu X, Lei X, Mu W (2022) Study on efficient and synergistic leaching of valuable metals from spent lithium iron phosphate using the phosphoric acid-oxalic acid system. Sep Purif Technol 303:122247. 10.1016/j.seppur.2022.122247

[CR32] Li Z, Zheng Q, Nakajima A, Zhang Z, Watanabe M (2023) Organic acid-based hydrothermal leaching of LiFePO4 cathode materials. Adv Sustainable Syst 1–12:2300421. 10.1002/adsu.202300421

[CR33] Lide, D.R. (2004) CRC Handbook of Chemistry and Physics, 84th Edition Edited by David R. Lide (National Institute of Standards and Technology). CRC Press LLC: Boca Raton. 2003. 2616 pp. $139.95. ISBN 0–8493–0484–9., Journal of the American Chemical Society. American Chemical Society (ACS). 10.1021/ja0336372

[CR34] Liu G, Liu Z, Gu J, Yuan H, Wu Y, Chen Y (2023a) A facile new process for the efficient conversion of spent LiFePO4 batteries via (NH4)2S2O8 -assisted mechanochemical activation coupled with water leaching. Chem Eng J 471:144265. 10.1016/j.cej.2023.144265

[CR35] Liu K Wang M Zhang Q Xu Z Labianca C Komárek M Gao B DCW Tsang 2023 A perspective on the recovery mechanisms of spent lithium iron phosphate cathode materials in different oxidation environments J Hazard Mater 445 10.1016/j.jhazmat.2022.130502 10.1016/j.jhazmat.2022.130502 36493647

[CR36] Liu W, Li K, Wang W, Hu Y, Ren Z, Zhou Z (2022) Selective leaching of lithium ions from LiFePO4 powders using hydrochloric acid and sodium hypochlorite system. Can J Chem Eng. 10.1002/cjce.24617

[CR37] Mahandra H, Ghahreman A (2021) A sustainable process for selective recovery of lithium as lithium phosphate from spent LiFePO4 batteries. Resour Conserv Recycl 175:105883. 10.1016/j.resconrec.2021.105883

[CR38] Meng, F., Liu, Q., Kim, R., Wang, J., Liu, G., Ghahreman, A. (2020) Selective recovery of valuable metals from industrial waste lithium-ion batteries using citric acid under reductive conditions: Leaching optimization and kinetic analysis. Hydrometallurgy 191. 10.1016/j.hydromet.2019.105160

[CR39] Niu Y, Peng X, Li J, Zhang Y, Song F, Shi D, Li L (2023) Recovery of Li2CO3 and FePO4 from spent LiFePO4 by coupling technics of isomorphic substitution leaching and solvent extraction. Chinese J Chem Eng 54:306–315. 10.1016/j.cjche.2022.04.005

[CR40] Saju D, Ebenezer J, Chandran N, Chandrasekaran N (2023) Recycling of lithium iron phosphate cathode materials from spent lithium-ion batteries : a mini-review. Ind Eng Chem Res 62:11768–11783. 10.1021/acs.iecr.3c01208

[CR41] Qin X, Yang G, Cai F, Wang B, Jiang B, Chen H, Tan C (2019) Recovery and Reuse of Spent LiFePO_4_ Batteries. J New Mat Electr Syst 22:119–124

[CR42] Sattar R, Ilyas S, Bhatti HN, Ghaffar A (2019) Resource recovery of critically-rare metals by hydrometallurgical recycling of spent lithium ion batteries. Sep Purif Technol 209:725–733. 10.1016/j.seppur.2018.09.019

[CR43] Schiavi PG, Altimari, P., Branchi, M., Zanoni, R., Simonetti, G., Navarra, MA., Pagnanelli, F. (2021) Selective recovery of cobalt from mixed lithium ion battery wastes using deep eutectic solvent. Chem Eng J 417 10.1016/j.cej.2021.129249

[CR44] Song S, Liu R, Sun M, Zhen A (2023) Hydrometallurgical recovery of lithium carbonate and iron phosphate from blended cathode materials of spent lithium-ion battery. Rare Met. 10.1007/s12598-023-02493-9

[CR45] Song Y, Xie B, Song S, Lei S, Sun W, Xu R, Yang Y (2021) Regeneration of LiFePO4 from spent lithium-ion batteries: via a facile process featuring acid leaching and hydrothermal synthesis. Green Chem 23:3963–3971. 10.1039/d1gc00483b

[CR46] Sun X, Hao H, Hartmann P, Liu Z, Zhao F (2019) Supply risks of lithium-ion battery materials: An entire supply chain estimation. Mater Today Energy 14:100347. 10.1016/j.mtener.2019.100347

[CR47] Takahashi VCI, Bothelho ABJ, Espinosa DCR, Tenório JAS (2020) Enhancing cobalt recovery from Li-ion batteries using grinding treatment prior to the leaching and solvent extraction process. J Environ Chem Eng 8:103801. 10.1016/j.jece.2020.103801

[CR48] Tang H Dai XI Li Q Qiao Y Tan F 2020 Selective Leaching of LiFePO4 by H2SO4 in the presence of NaClO3 Revista de Chimie 71 248 254 10.37358/RC.20.7.8242

[CR49] Tao S, Li J, Wang L, Hu L, Zhou H (2019) A method for recovering Li3PO4 from spent lithium iron phosphate cathode material through high-temperature activation. Ionics (kiel) 25:5643–5653. 10.1007/s11581-019-03070-w

[CR50] Vieceli N, Casasola G, Lombardoebin G, Ebin B, Petranikova M (2021) Hydrometallurgical recycling of EV lithium-ion batteries: Effects of incineration on the leaching efficiency of metals using sulfuric acid. Waste Manag 125(192):203. 10.1016/j.wasman.2021.02.039 10.1016/j.wasman.2021.02.039 33706256

[CR51] Wang XJ, Zheng SL, Zhang Y, Zhang Y, Qiao S, Long ZQ, Zhao B, Li ZF (2022a) Sulfuric acid leaching of ball-milling activated FePO4 residue after lithium extraction from spent lithium iron phosphate cathode powder. Waste Manag 153:31–40. 10.1016/j.wasman.2022.08.009 36049270 10.1016/j.wasman.2022.08.009

[CR52] Wang M Liu K Dutta S Alessi DS Rinklebe J Ok YS DCW Tsang 2022 Recycling of lithium iron phosphate batteries: status, technologies, challenges, and prospects Renew Sustain Energy Rev 163 10.1016/j.rser.2022.112515

[CR53] Wang YH, Wu JJ, Hu GC, Ma WH (2023) Recovery of Li, Mn, and Fe from LiFePO4/LiMn2O4 mixed waste lithium-ion battery cathode materials. J Min Metall Sect B Metall 59:17–26. 10.2298/JMMB220918002W

[CR54] Wood Mackenzie (2020) Can LFP technology retain its battery market share? Report | Wood Mackenzie URL https://www.woodmac.com/reports/power-markets-can-lfp-technology-retain-its-battery-market-share-428028/ Accessed 11 Oct 2023

[CR55] Wu L, Zhang FS, Zhang ZY, Zhang CC (2023) An environmentally friendly process for selective recovery of lithium and simultaneous synthesis of LiFe5O8 from spent LiFePO4 battery by mechanochemical. J Clean Prod 396:136504. 10.1016/j.jclepro.2023.136504

[CR56] Xu C, Hu X, Yang Y, Jian Z, Chen W, Yang L (2023) Integrated process of CO2 sequestration and recycling spent LiFePO4 batteries. Energy Storage Mater 60:102819. 10.1016/j.ensm.2023.102819

[CR57] Yadav P, Jie CJ, Tan S, Srinivasan M (2020) Recycling of cathode from spent lithium iron phosphate batteries. J Hazard Mater 399:123068. 10.1016/j.jhazmat.2020.123068 32521319 10.1016/j.jhazmat.2020.123068

[CR58] Yagci MC Behmann R Daubert V Braun JA Velten D Bessler WG 2021 Electrical and structural characterization of large-format lithium iron phosphate cells used in home-storage systems Energy Technol 9 10.1002/ente.202000911

[CR59] Yang Y, Meng X, Cao H, Lin X, Liu C, Sun Y, Zhang Y, Sun Z (2018) Selective recovery of lithium from spent lithium iron phosphate batteries: A sustainable process. Green Chem 20:3121–3133. 10.1039/c7gc03376a

[CR60] Yang Y, Sun M, Yu W, Ma X, Lei S, Sun W (2023) Recovering Fe, Mn and Li from LiMn1-xFexPO4 cathode material of spent lithium-ion battery by gradient precipitation. Sustain Mater Technol 36:e00625. 10.1016/j.susmat.2023.e00625

[CR61] Yang Y, Zhang J, Zhang H, Wang Y, Chen Y (2024) Simultaneous anodic de-lithiation / cathodic lithium-embedded regeneration method for recycling of spent LiFePO4 battery. Energy Storage Mater 65:103081. 10.1016/j.ensm.2023.103081

[CR62] Yue Y, Wei S, Yongjie B, Chenyang Z, Shaole S, Yuehua H (2018) Recovering valuable metals from spent lithium ion battery via a combination of reduction thermal treatment and facile acid leaching. ACS Sustain Chem Eng 6(8):10445–10453. 10.1021/acssuschemeng.8b01805

[CR63] Zeng X, Li J, Shen B (2015) Novel approach to recover cobalt and lithium from spent lithium-ion battery using oxalic acid. J Hazard Mater 295:112–118. 10.1016/j.jhazmat.2015.02.064 25897692 10.1016/j.jhazmat.2015.02.064

[CR64] Zhang L, Teng T, Xiao L, Shen L, Ran J, Zheng J, Zhu Y, Chen H (2022) Recovery of LiFePO4 from used lithium-ion batteries by sodium-bisulphate-assisted roasting. J Clean Prod 379:134748. 10.1016/j.jclepro.2022.134748

[CR65] Zhang Q, Fan E, Lin J, Sun S, Zhang X, Chen R (2023a) Acid-free mechanochemical process to enhance the selective recycling of spent LiFePO4 batteries. J Hazard Mater 443:130160. 10.1016/j.jhazmat.2022.130160 36283216 10.1016/j.jhazmat.2022.130160

[CR66] Zhang Q, Fan E, Lin J, Sun S, Zhang X, Chen R (2023b) Acid-free mechanochemical process to enhance the selective recycling of spent LiFePO_4_ batteries 443. 10.1016/j.jhazmat.2022.130160 10.1016/j.jhazmat.2022.130160 36283216

[CR67] Zhao S, Quan J, Wang T, Song D, Huang J, He W, Li G (2022) Unveiling the recycling characteristics and trends of spent lithium - ion battery : a scientometric study. Environ SciPollut Res 9448–9461 10.1007/s11356-021-17814-7 10.1007/s11356-021-17814-7 34855174

[CR68] Zheng R, Zhao L, Wang W, Liu Y, Ma Q, Mu D, Li R, Dai C (2016) Optimized Li and Fe recovery from spent lithium-ion batteries: via a solution-precipitation method. RSC Adv 6:43613–43625. 10.1039/c6ra05477c

[CR69] Zhou H, Luo Z, Wang S, Ma X, Cao Z (2023a) A mild closed-loop process for lithium – iron separation and cathode materials regeneration from spent LiFePO4 batteries. Sep Purif Technol 315:123742. 10.1016/j.seppur.2023.123742

[CR70] Zhou H, Zhang Y, Li L, Cao Z (2023b) Integrated recycling of valuable elements from spent LiFePO_4_ batteries: a green closed-loop process. 7696–7706. 10.1039/d3gc02180g

